# The emerging role of nuclear receptor coactivator 4 in health and disease: a novel bridge between iron metabolism and immunity

**DOI:** 10.1038/s41420-024-02075-3

**Published:** 2024-07-03

**Authors:** Yue Le, Qinjie Liu, Yi Yang, Jie Wu

**Affiliations:** 1 Jiangsu Provincial Key Laboratory of Critical Care Medicine, Department of Critical Care Medicine, Zhongda Hospital, School of Medicine, Southeast University, Nanjing, 210009 China; 2 Department of General Surgery, Zhongda Hospital, School of Medicine, Southeast University, Nanjing, 210009 China; 3 Research Center of Surgery, BenQ Medical Center, the Affiliated BenQ Hospital of Nanjing Medical University, Nanjing, 210021 China

**Keywords:** Cell death, Cell signalling, Cell death and immune response

## Abstract

Nuclear receptor coactivator 4 (NCOA4) has recently been recognized as a selective cargo receptor of ferritinophagy participating in ferroptosis. However, NCOA4 is also a coactivator that modulates the transcriptional activity of many vital nuclear receptors. Recent novel studies have documented the role of NCOA4 in healthy and pathogenic conditions via its modulation of iron- and non-iron-dependent metabolic pathways. NCOA4 exhibits non-ferritinophagic and iron-independent features such as promoting tumorigenesis and erythropoiesis, immunomodulation, regulating autophagy, and participating in DNA replication and mitosis. Full-length human-NCOA4 is composed of 614 amino acids, of which the N-terminal (1–237) contains nuclear-receptor-binding domains, while the C-terminal (238–614) principally contains a ferritin-binding domain. The exploration of the protein structure of NCOA4 suggests that NCOA4 possesses additional significant and complex functions based on its structural domains. Intriguingly, another three isoforms of NCOA4 that are produced by alternative splicing have been identified, which may also display disparate activities in physiological and pathological processes. Thus, NCOA4 has become an important bridge that encompasses interactions between immunity and metabolism. In this review, we outline the latest advances in the important regulating mechanisms underlying NCOA4 actions in health and disease conditions, providing insights into potential therapeutic interventions.

## Facts

- NCOA4 affects diverse physiological or pathological conditions by orchestrating iron metabolism.
- As a nuclear receptor coactivator, NCOA4 functions in various metabolic pathways.
- NCOA4 regulates immune responses via iron metabolism-dependent and -independent mechanisms.
- The NCOA4-targeted intervention has shown therapeutic potential.

## Open questions

- What is the specific molecular mechanism underlying the expression and regulation of immunometabolism by NCOA4 in both physiological and pathological conditions?
- Are there any other post-translational modifications of NCOA4, and how do these modifications regulate its bioactivity?
- Can NCOA4 sense and regulate the metabolism of other similar trace elements?

## Introduction

Nuclear receptor coactivator 4 (NCOA4) was recently discovered as a specific cargo receptor for ferritin, which selectively deliver ferritin to autophagosomes (ferritinophagy) and began to attract attention of investigations [1]. In 2016, two independent groups reported the role of NCOA4-mediated ferritinophagy in ferroptosis [2, 3]. NCOA4 also displays an essential function in iron-related physiological functions including iron homeostasis, metabolism, transport, release, and utilization, by regulating the level of the intracellular labile iron pool (LIP). However, the impact of NCOA4 in regulating iron metabolism is not limited to cell death. Recent studies have shown that NCOA4 participates in a variety of important physiological and pathological processes and mediates the onset and progression of multiple diseases via its highly conserved structure domains and function [4–8].

NCOA4 was originally discovered as a component of ret fused gene by Santoro et al. [9] in 1994 and is prominently expressed in human papillary thyroid carcinomas. In normal tissue, NCOA4 is highly expressed in both reproductive and non-reproductive tissues of adult mice, including the adrenal gland, prostate, testis, heart, intestine, spleen, lung, and kidney, while being undetectable in the brain cortex [10, 11]. Moreover, NCOA4 is expressed ubiquitously and dynamically during embryonic development [11].

Interestingly, NCOA4 is involved in modulating both innate and acquired immunity responses resulting from iron regulation function or interaction with specific proteins [12–14]. In 1996, Yeh et al. [10] first reported gene-regulatory function of NCOA4, indicating that NCOA4 interacts with the androgen receptor (AR) and enhances its transcriptional activity. Initially, NCOA4 was named as androgen receptor-associated protein of 70 kDa (ARA70). More recent studies have revealed that the full length of human NCOA4 consists of 614 amino acid residues [15] comprising the N-terminal coiled-coil domain (contains conserved ARA70 domain and other nuclear receptors binding regions) and a C-terminal domain known as ferritin-binding domain (FBD) [10]. NCOA4 also interacts with other nuclear receptors such as the steroid receptor superfamily (including glucocorticoid receptor [GR], estrogen receptor [ER], progesterone receptor [PR]) and non-steroidal receptor (thyroid hormone receptor [TR]), vitamin D receptor [VDR], peroxisome proliferator-activated receptor [PPAR] and aryl hydrocarbon receptor (AHR) [16], regulating their transcriptional activity.

Thus, beyond the canonical role of NCOA4 in ferritinophagy and ferroptosis, growing evidence highlights its novel emerging role in metabolism and immunity. In this review, we summarize the important roles of NCOA4 in metabolism and immunity and discuss the non-ferritinophagic features and precise regulation of NCOA4 under physiological or pathological conditions. We also summarize the latest transformation medicine evidence of targeting NCOA4.

## The roles of NCOA4 in iron metabolism

Iron is the central transition metal in almost all living organisms [17]. Ferric iron is stable in the presence of oxygen but insoluble, whereas ferrous iron serves as an electron donor with good solubility in aqueous solutions. Due to those chemical properties, numerous biochemical processes rely on ferrous iron [17], including oxygen binding and transport [18], ATP production (as a cofactor in the citric acid cycle and electron transport) [19], and DNA biosynthesis and repair [20, 21]. However, ferrous iron is also potentially toxic because of its chemical properties. Thus, the iron level in living cells is tightly regulated by establishing a balance between iron validity and retention. Recent studies identified the critical role of NCOA4 in maintaining iron homeostasis, facilitating the sensing and modulating of intracellular labile iron (Fe(II)) [1, 22, 23]. This section discusses the mechanisms of NCOA4 in orchestrating iron metabolism under physiological and pathological conditions.

### Regulation roles of NCOA4 in orchestrating iron homeostasis

**Fig. 1:**
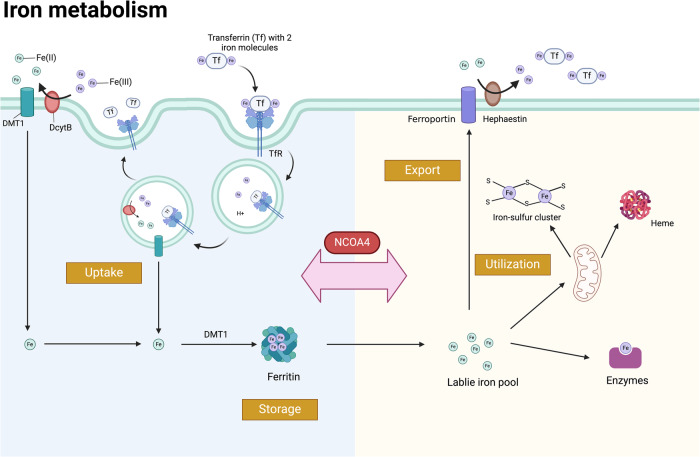
The main regulation pathway of intracellular iron metabolism. The iron metabolism in vivo consists of uptake, utilization, storage, and export. Duodenal cytochrome B (DcytB) restores ferric iron as ferrous iron, the latter then is transported into the cytoplasm via divalent metal-ion transporter-1 (DMT1). As a main form of circulating iron, transferrin (Tf), is responsible for transporting ferric iron via internalization in an endosome pathway after being recognized by the TfR. Then the TfR1-TF complex is processed back to the cell surface for further reuse after iron releasing to cytoplasm. The intracellular labile iron pool is usually used for (i) the synthesis of heme/hemoglobin and iron-sulfur cluster in mitochondria; (ii) it acts as a cofactor for multiple enzymes. Overload iron is combined with ferritin for storage or exported from cells by ferroportin. The output ferrous iron is oxidized by Hephaestin, binding to Tf for circulating transportation. NCOA4 is vital in the mutual transformation between storage iron (ferritin) and the labile iron pool. The Fe(II) is represented in green circle, and the Fe(III) is represented in purple circle shape. The figure is created with BioRender.com.

In normal cells, iron uptake, utilization, storage, and export are carefully coordinated so that the pool of labile iron (Fe(II)) remains relatively stable (Fig. 1). Intracellular iron is mainly stored in ferritin, and Fe (III) must be reduced and released from ferritin for utilization [24]. As early as 2009, Di Domenico et al. [25] reported two routes for ferritin degradation: deferoxamine (DFO)-induced autophagy under iron depletion and cytosolic degradation via proteasome after the release of iron. Asano et al. [26] proposed the mechanism for the release of iron, in which ferritin is delivered to the lysosome in primary cells by autophagy under iron-depleted or autophagy-independent pathways in iron-replete conditions. In contrast, in cancer cells, excess iron is still stored by ferritin even under iron-rich conditions without ferritin degradation. This partly explained why some cancer cells are resistant to the high level of iron and suggested that there was also a certain key mechanism that regulates ferritin degradation in various cells.

In 2014, two groups identified the novel role of NCOA4 in ferritin autophagy under conditions of iron depletion [1, 22]. Dowdle et al. [22] identified that NCOA4 interacted with the ferritin heavy chain (FTH1) but not the ferritin light chain (FTL), thus regulating the turnover of ferritin by autophagy, which relies on the vacuolar protein sorting (VSP)34-mediated LC3 lipidation. In parallel, Mancias et al. [1] identified NCOA4 as a selective cargo receptor for the autophagic turnover of ferritin under iron-depleted conditions. Then further revealed that ferritin-binding residues I489/W497 of NCOA4 and R23 of FTH1 are essential for their binding and are required for ferritinophagy [23]. In addition, the domain for HECT and RLD domain containing E3 ubiquitin protein ligase 2 (HERC2), a type of E3 ubiquitin-protein ligase, binding on NCOA4 overlaps with the FTH1 binding site; NCOA4 combined with iron provides a targeted for ubiquitylation by HERC2, leading to NCOA4 degradation via the proteasome pathway under iron-repletion conditions [23]. Furthermore, an independent study elaborates on the interaction between NCOA4 and ferritin based on the thermodynamic theory. It was demonstrated that approximately eight NCOA4 molecules specifically bind an H-rich ferritin shell, and FTH1 can bind up to 24 NCOA4 fragments [27]. Similarly, the binding reaction of FTH1 and NCOA4 was demonstrated to be both enthalpically and entropically favored, the iron release kinetics was superior to NCOA4-FTH1 complexes, and iron release from ferritin was inhibited by NCOA4 in a concentration-dependent manner [28].

**Fig. 2:**
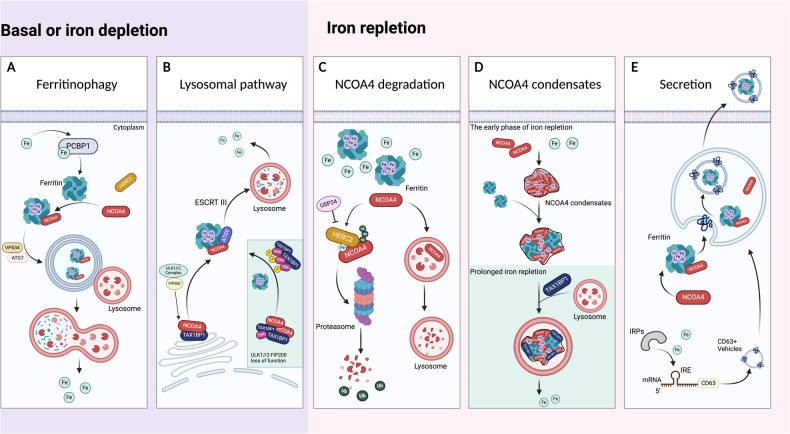
Regulation of NCOA4 in orchestrating intracellular iron homeostasis. Upon the basal or iron depletion conditions, two main mechanisms regulate the cellular iron level (**A**, **B**): (**A**) *Classical ferritinophagy pathway*. NCOA4 interacts with ferritin-heavy chain (FTH)1, transferring autophagosomes to lysosomes to degrade ferritin and release free iron. VPS34-mediated LC3 lipidation and ATG7 involve in ferritinophagy. **B** *Autophagy-independent lysosomal targeting regulated pathway*. The ULK1/2-FIP200 complex regulates the dissociation of NCOA4 from TAX1BP1. ATG9, VPS34, and ESCRT-III are necessary for the trafficking of NCOA4-ferritin complexes to lysosomes to release free iron. ULK1/2-FIP200 loss of function results in aggregates of TAX1BP1-NCOA4. Instead, TAX1BP1 recruits TBK1 to regulate basal ferritin flux. Upon the iron repletion conditions, the main mechanisms that modulate the cellular iron level were shown in (**C**–**E**): (**C**) *NCOA4 degradation via proteasome and lysosome pathways*. NCOA4 combined with iron provides a target for HERC2 ubiquitination of NCOA4, which is recognized and degraded by proteasomes. NCOA4 can also be directly degraded by lysosomes. **D** *NCOA4 forms condensate via binding with Fe (III) in the early phase of iron repletion*. The NCOA4 condensates sequester ferritin, forming a complex for accumulation to avoid iron overload. During prolonged iron supply, TAX1BP1 binds NCOA4 and delivers ferritin to lysosomes to prevent iron deficiency. **E** *NCOA4 regulates the secretion of ferritin in a CD63-dependent pathway*. Iron repletion induces IRP dissociating from the IRE of CD63 mRNA, initiating the translation of CD63, increasing the CD63 expression in extracellular vehicles (EVs), as well as the secretion of CD63-EVs that contains ferritin.

To ensure the availability of labile iron while circumventing peroxidation toxicity under various conditions, there are multiple pathways for NCOA4-related ferritin degradation except for NCOA4-mediated ferritinophagy (Fig. 2). Recently, Kuno et al. [29] revealed that NCOA4 also plays vital roles in determining the fate of ferritin under iron-repletion conditions. Intrinsically disordered regions (IDR), together with the C-terminal of NCOA4, participated in the formation of an insoluble condensate via the binding of Fe(III) to generate multivalent interactions. This sequesters NCOA4 away from ferritin to form NCOA4 condensates with an approximately diameter of 120 nm and maintains ferritin stabilization in the early phase of iron repletion. As repletion time is prolonged, tax1-binding protein 1 (TAX1BP1) acts as a receptor for aggrephagy, binding to NCOA4 via its aa 446–484 region, then delivering ferritin to lysosomes in an autophagy-related protein (ATG) 7-independent fashion, thereby preventing iron deficiency caused by excessive iron storage [29]. Therefore, NCOA4 is more likely an iron sensor and oxidation reaction detector to regulate ferritin fate in accordance with the ferric ion level. However, the relation between TAX1BP1 and HERC2 in modulating the level of NCOA4 still requires further investigation.

In addition, Goodwin et al. [30] found an alternative lysosomal transport pathway for the degradation of ferritin that requires FIP200, ATG9A, VPS34, and TAX1BP1 by using genome-scale functional screening. Under basal and iron-depleted conditions, TAX1BP1, but not ATG8, interacts with NCOA4 and is involved in the lysosomal trafficking of ferritin. The loss of function of ULK1/2-FIP200 impaired the ferritin trafficking from Golgi to the lysosome, leading to cytosolic aggregates of TAX1BP1 and NCOA4. TAX1BP1 recruits TANK-binding kinase 1 to regulate the redistribution of ATG9A to the Golgi, compensating basal ferritin flux to increase the level of cellular iron [30]. These findings suggest that the level of cellular iron is precisely controlled by a complex system involved in NCOA4, which is regulated and changed accordingly in specific cell types and various stress conditions. However, further studies are required to explore the mechanism of the ferritin degradation pathway during different conditions, such as hyperglycemia, hypertonicity, and hyperpyrexia.

Intriguingly, some investigators have reported a novel role of NCOA4 in orchestrating the secretion of iron or ferritin. Previous studies have suggested that ferroportin is the primary exit pathway and the only known ferrous iron transporter [31]. However, in recent years, secreted ferritin has been suggested as an iron exporter. Cohen LA et al. [32] found serum ferritin is secreted by splenic macrophages and proximal tubule cells of the kidney rather than leaking from damaged cells in a murine model. However, mammalian ferritin is a cytoplasmic protein lacking the signal peptide for the conventional secretory pathway (endoplasmic reticulum-Golgi secretion). Meyron-Holtz and colleagues [31] revealed that both the non-classical secretory-autophagy pathway and multivesicular-body-exosome pathway were involved in ferritin secretion in mouse models of impaired endo-lysosomal trafficking. Further investigations reported that knockdown or knockout of NCOA4 did not affect the level of serum ferritin, even increased the secretion of ferritin under blocked endo-lysosomal trafficking or damaged lysosomal conditions [24, 31, 33], which suggests that NCOA4 does not take part in secretory autophagy of ferritin. However, recent studies uncovered a novel mechanism by which NCOA4 is responsible for transferring ferritin to CD63 for secretion under cellular iron loading conditions. The expression of CD63, an extracellular vesicle-associated protein, is under the regulation of the iron regulatory element (IRE)- iron responsive protein (IRP) system, which is activated in response to increased iron [34]. Likewise, another independent research group also demonstrated that chloroquine (CQ), a lysosomal acidification inhibitor, induces the release of autophagy receptors, including NCOA4, via single membrane endosomes and double-membrane compartment pathways [35]. These findings suggested that NCOA4 possesses the ability to enhance secretion in certain stress condition, whereas compensatory mechanisms take over the function as NCOA4 is deficiency prolonged. Further investigation will be necessary to explicit the NCOA4-related trafficking during the acute phase and chronic transition phase, which may be important for figuring out the roles of NCOA4 in the local cell-to-cell exchange of iron.

### NCOA4 supports erythropoiesis

Given the pivotal roles of NCOA4 in iron metabolism as described above, several studies have revealed its physiological function in erythropoiesis which aims to generate enough red blood cells for the host. In 2005, Weber et al. [36] first found the biological connection between NCOA4 and erythropoiesis in a zebrafish model--NCOA4 is a novel erythroid transcription factor that was identified as a late erythroid marker. The enhanced expression of NCOA4 gene in the erythroid lineage was also identified through a large-scale gene transcriptome analysis [37]. Erythropoiesis is tightly regulated by NCOA4 via sustaining the availability of labile iron.

Ryu et al. has elaborated on the role of NCOA4 during the whole process of erythropoiesis via directing iron release and trafficking [38]. At the beginning of terminal differentiation, the expression of NCOA4 remains low, heme and hemoglobin synthesis have not begun. During the metaphase, polyC-binding protein 1 (PCBP1) carries Fe(II) and delivered it to ferritin, which is selectively captured and mediated into the autophagosome by NCOA4. Subsequently, the ferritin is transferred to the lysosome for dissolution, releasing ferrous iron to synthesize heme in mitochondria until late erythrocyte development while iron is not directed into ferritin. Thus, NCOA4 plays a role in the mobilization of iron when heme synthesis occurs.

Interestingly, the regulation of erythropoiesis by NCOA4 showed significant tissue differences. Systemic NCOA4 knockout mice developed more severe anemia that disrupted general iron homeostasis, resulting in the accumulation of tissue ferritin and iron, a reduction in serum iron content, and anemia. Mice with erythroid-specific ablation showed severe anemia postnatally but hypochromic microcytic anemia in adulthood, indicating that NCOA4 is required for erythroid differentiation in postnatal mice rather than in adult mice [39]. Erythroid intrinsic NCOA4 function in differentiation may be compensated by hypoxia-inducible factors (HIF)2α-erythropoietin (EPO) system [39, 40]. The major tissue for iron storage in mammals is liver, in which hepatocytes accumulate iron within ferritin; Li et al. [41] established a hepatocyte-targeted NCOA4 knockdown murine model in which they induced acute iron deficiency and stress erythropoiesis with phlebotomy, and they demonstrated a markedly impaired ability to degrade ferritin or mobilize iron in hepatocytes of NCOA4^−/−^ mice, which revealed that during acute iron deficiency, NCOA4-mediated turnover of ferritin plays a role in iron mobilization from the liver.

The contribution of NCOA4-mediated ferritinophagy in macrophages thus cannot be ignored. Inactivation of NCOA4 increases cellular ferritin and iron accumulation in organs, tissues, and cells, in particular splenic macrophages [22, 33]. Nai. A et al. [42] proved that the decline of iron release by macrophages was the principal driving factor of anemia in NCOA4^−/−^ animals, especially those manifesting iron deficiencies; however, whether this phenomenon is caused by an inherent defect of erythrocytes or iron retention due to ferritin degradation dysfunction caused by inactivation of NCOA4 remains to be further studied. Most studies evaluate the role of NCOA4 in erythropoiesis (but under a conditional knockout), the host can develop compensatory mechanisms that maintain hemoglobin synthesis with NCOA4 loss in erythroid or other cells during the chronic phase, which may conceal the function of NCOA4. A conditional NCOA4-knockout mouse using an inducible Cre-lox system may therefore be necessary.

### NCOA4 modulates ferroptosis via mediating ferritinophagy

Both iron depletion and overload can lead to diseases; with the latter is associated with a type of programmed cell death uncovered only recently. Ferroptosis is a novel form of programmed cell death first described by Dixon et al. [43] in 2012 and is typically characterized by iron-dependent lethal lipid peroxidation. The essence of ferroptosis is due to the iron overload catalyzing the accumulation of reactive oxygen species (ROS) on the membrane lipid mainly via the Fenton reaction, leading to an imbalance of redox in cells and inducing cell death. Additionally, it is accompanied by the suppression of the cystine/glutamate transporter system Xc and antioxidase activities such as by GPX4 [44].

An imbalance of iron metabolism may lead to intracellular iron overload. The degradation of ferritin is also one of the chief mechanisms for regulating the LIP. It has been demonstrated that ferritinophagy leads to ferritin degradation and the release of ferrous iron into the LIP, enhancing the sensitivity of ferroptosis [2, 3]. NCOA4, which occupies a central role in regulating intracellular iron levels, has been found to modulate the sensitivity of ferroptosis [2]. As such, the knockdown of NCOA4 elevated FTH1 expression and limited erastin-induced cell death, whereas overexpression of NCOA4 increased intracellular Fe [45] and malondialdehyde, which suggested NCOA4-mediated ferritin degradation is involved in ferroptosis [43]. Gao et al. demonstrated that the NCOA4-mediated ferritin degradation increased the level of LIP—resulting in ferroptosis [3]—and emphasized the influence of inhibiting autophagy on ferroptosis is more obvious at the early stage of ferroptosis, which is in line with the result from Hou et al. [2]. It was also demonstrated that autophagy-induced ferroptosis through ferritin degradation was dependent on ATG5 and ATG7. In our previous work, we identified NCOA4-activated ferritinophagy as under the regulation of the stimulator of the interferon response cGAMP interactor 1 (STING) pathway in accelerating macrophage ferroptosis. Our work revealed an interaction between NCOA4 and immune response, suggesting a potential role for NCOA4 in immune regulation [14]. Further studies are still required to address the regulation of autophagy/ferritinophagy as it relates to ferroptosis.

## The roles of NCOA4 as a co-regulator of nuclear receptor

NCOA4 is known to be an early coactivator of the AR. Investigators have identified two ARA70 family functional domains in NCOA4, located at amino acids 37–167 (ARA70-I) and 138–332 (ARA70-II) [46]. NCOA4 is evolutionarily conserved; orthologs of NCOA4 can be found in various metazoans. NCOA4 enhances the transactivation of AR induced by ligands other than active androgens when overexpressed [16]. Similarly, NCOA4 can interact with non-steroidal nuclear receptors such as TR and steroid hormone receptors, including ER, PR, AHR, and VDR [47–50]. Kollara et al. [16] have already described the metabolism-related functions of NCOA4 on these nuclear receptors in an excellent review; thus, here we focus on TR and PPARs.

The thyroid hormone T3 plays a substantial role in cellular development, differentiation, and metabolism; and its actions are mediated by nuclear TRs [51]. One study revealed that NCOA4 regulates the expression of erythroid differentiation genes in response to thyroid hormone stimulation [52], and erythroid terminal differentiation in mature erythroblasts can be impaired if NCOA4 is knockdown. ChIP-seq analysis showed a closer chromatin binding site for NCOA4 to Pol II after GC-1 treatment (a type of TRβ agonist that can be used to enhance erythroid differentiation). This recruitment promotes the regulation of the expression of the erythroid genes in response to TH signaling [52]. These functions are complementary to iron metabolism in regulating erythropoiesis. Thus, targeting the coordination of NCOA4 and TR shows potential for treating certain anemias.

**Fig. 3:**
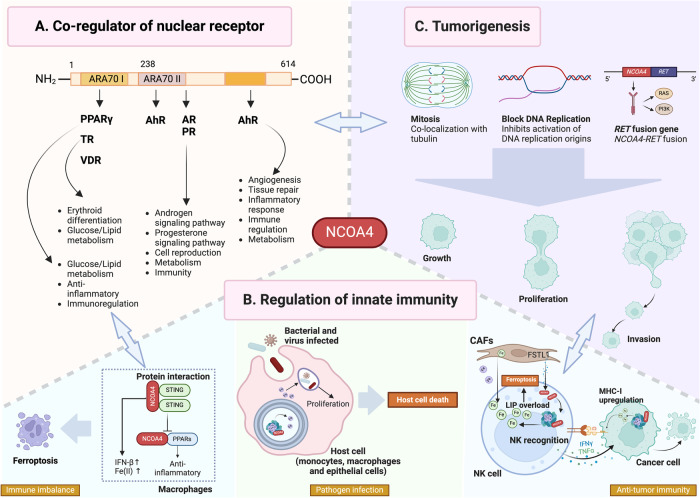
Other functions of NCOA4 under physiological or pathological conditions. **A** *NCOA4 is a co-regulator of nuclear receptors*. Conserved ARA70 domain I (amino acids 37–167) contains a specific motif interacting with PPARγ, TR, and VDR. ARA70 domain II (amino acid 138–332) interacts with AR, PR, and AHR. The interaction between NCOA4 and these nuclear receptors regulates corresponding physiological and pathological processes. **B** *Novel roles of NCOA4 in innate immunity regulation*. NCOA4 exerts its anti-tumor, anti-infection, and immunoregulation functions by orchestrating iron metabolism and interacting with proteins. **C** *NCOA4 in tumorigenesis*. The NCOA4-RET fusion shows stronger invasiveness in tumors. NCOA4-RET also participates in cell growth, proliferation, and invasion via regulating the initiation of DNA replication, co-localization with tubulin, and mitosis, without clear cognition of molecular mechanism. Abbreviation: CAFs cancer associated fibroblasts, FSTL1 follistatin like protein 1.

Recent results suggest that PPARs are core links between metabolic lipid diseases and immune homeostasis [53]. PAR-γ is involved in various biological processes, acting as a critical transcriptional regulator for fatty acid and glucose metabolism, while also contributing to the anti-inflammation in innate immune response [54]. Previous studies have identified NCOA4 as a ligand-enhanced coactivator for PPARγ, enhancing the transcription activity of PPARγ. Subsequent research has found that decreased expression of NCOA4 alleviates the activity of PPARγ in C. difficile-infected mice [12], indirectly confirming the functions of this coactivator of NCOA4. Another subtype of PPAR, PPAR-α, is highly expressed in the liver, kidney, adrenal gland, and male and female reproductive systems; and NCOA4 has been reported to enhance the transcriptional activity of PPARα, while its coactivation function was attenuated with a point mutation in the PPARα ligand-binding domain [55]. Therefore, regulation of NCOA4 may also show great therapeutic potential in anti-inflammatory and metabolic modulation (Fig. 3).

## NCOA4 in regulating the immune system

It was found that NCOA4 is involved in anti-infection and anti-tumor immunity via an influence of the level of iron or interaction with specific proteins. Sottile et al. [13] confirmed that NCOA4 knockout caused an accumulation of FTH1 and impaired ferritinophagy, increasing the expression of MHC class I on cell surfaces. The down-modulation of iron concentration allows natural killer (NK) cells to sense iron fluctuations via alterations in the MHC expression of its target cells, inducing a highly susceptible immune phenotype of NK cells. Recently, Yao et al. [56] reported the cancer associated fibroblasts-derived follistatin like protein 1 upregulates NCOA4 expression in NK cells responsible for NK cell ferroptosis, which result in an impair anti-tumor capacity of NK cells. These results suggest a role for NCOA4 in regulating the immune system in anti-cancer by affecting the metabolism of the host.

Iron is an indispensable nutrient for host homeostasis and pathogen replication. Hence, NCOA4 also influences the anti-infection response. Bauckmana et al. [57] demonstrated that uropathogenic E. coli persist within bladder epithelial cells by regulating ferritinophagy. Likewise, in Ehrlichia chaffeensis (an intracellular bacterium [58]) -infected host monocytes and macrophages, Ehrlichia translocated factor-3 is secreted into the cytoplasm [59], binding FTL and inducing ferritinophagy by recruiting NCOA4 and LC3; this increases the cellular LIP for feeding Ehrlichia. Similarly, *Mycobacterium* tuberculosis promoted NCOA4-mediated ferritin degradation through TRIM21-mediated proteasomal degradation of HERC2, enabling its intracellular survival [5]. In virus infection, human cytomegalovirus protein pUL38 binds to host protein ubiquitin-specific protease 24 (USP24), inhibiting NCOA4-mediated ferritinophagy against host fibroblasts cell death [60]. These findings suggest that NCOA4 is involved in the infection process by interacting with some pathogenic components. However, the mechanism of NCOA4 in various infections needs to be clarified to precisely target the treatment of infectious diseases.

As mentioned above, NCOA4 may regulate PPAR-mediated anti-infection immunity. Viladomiu et al. [12] found that down-regulated NCOA4 due to an overexpression of miRNA146b suppresses the activity of PPARγ and aggravates inflammatory responses in the colon of C. difficile-infected mice. Notably, in our recent study, we reported that NCOA4 regulated the innate immune system by directly interacting with STING. The STING pathway is widely distributed in immune cells, especially in macrophages, leading to sepsis organ damage after activation [61]. Our work showed that an interaction between STING and NCOA4 reduced NCOA4 nuclear localization, impairing the function of NCOA4 as a transcription factor co-regulator of PPAR, and resulting in decreased expression of PPAR downstream target genes [14]. This interaction also stabilized the STING dimer, enhancing the immune cascade pathway and inducing ferroptosis in macrophages during sepsis. Wang et al. [62] demonstrated that hydrogen sulfide alleviated particulate matter-induced airway inflammation by suppressing NCOA4-mediated ferritinophagy and ferroptosis, but that PPARγ inhibitor pretreatment significantly enhanced ferroptotic injury, revealing a vital link between the PPARγ-signaling pathway and anti-ferroptosis. Nevertheless, with respect to anti-tumor immunity, PPARγ-induced dendritic cell ferroptosis damages cell maturation and tumor suppression [63]. All the above findings provide us with additional insights into the mechanism underlying NCOA4’s involvement in immune regulation and the potential treatment of immune-related diseases such as inflammatory disorders, tumor immunity, and autoimmune diseases (Fig. 3).

Thus, NCOA4 may be involved as an important molecule in anti-tumor and anti-infection immunity. Unfortunately, apart from macrophages and NK cells, there have been no reports on the immune function of NCOA4 in other immune cells despite its universal expression across various immune cell types.

## The roles of NCOA4 in tumorigenesis

Different NCOA4 isoforms exert disparate effects on tumor progression (Fig. 3). For example, a significant function of NCOA4b (which lacks the FBD domain compared with full-length NCOA4a) was demonstrated in prostate cancer [64]. Genome-wide microarray analysis showed that the differential expression of 953 genes in LNCaP cells was influenced by NCOA4b compared with NCOA4a. Genes regulating cell adhesion were downregulated, whereas those involved in cell proliferation were upregulated, consistent with the tumorigenic effect of NCOA4b. This NCOA4 isoform is also differentially expressed in invasive breast cancers. While western-blot analysis of breast cancer tissue from patients exhibited attenuated NCOA4a expression and detectable NCOA4b, NCOA4a but not NCOA4b expression was observed in adjacent benign tissue [65]. Recently, Chan and his group [64] discovered a novel, nearly full-length NCOA4 isoform in rectal sessile serrated lesions, and *NCOA4-RET* injection induced non-tumorous cells to grow faster and transform into tumors within 14 days in athymic nude mice. Thus, the relationship between NCOA4 isoforms and tumor progression requires further elucidation.

Intriguingly, although authors previously reported a tumor-suppressor function of full-length NCOA4, in contrast, an AR-dependent promotion of NCOA4b was shown in prostate cancer cell growth and invasion, suggesting a role of full-length NCOA4 in regulating normal cell proliferation [64, 66]. Kollara et al. [46] observed co-localization of NCOA4 with α-, β-, and acetylated tubulin in non-mitotic cells and prophase mitotic cells, and co-localization of NCOA4 with the microtubule organizing center and actin was also observed during interphase. However, NCOA4 accumulation at the centrosomes was significantly reduced in mitotic metaphase. These findings suggest that NCOA4 contributes to microtubule nucleation and cytokinesis (cell division). The authors of one study depicted an NCOA4-dependent mechanism that coordinated intracellular iron bioavailability and DNA metabolism, protecting DNA from replicative stress damage [67].

In addition, application of an *NCOA4*-RET fusion gene suggests a critical role for the C-terminal of NCOA4 in modulating the activity of signaling pathways, as the *RET* oncogene encodes the transmembrane receptor of the tyrosine kinase family [9]. There are different forms of *RET* fusion. For example,Yang et al. [68] assessed several clinical researches in large pan-cancer cohorts, which found the most common partner of *RET* is *KIF5B* (45%), followed by *CCDC6* (29.1%) and *NCOA4* (13.3%). Intriguingly, Levinson et al. [69] found that *NCOA4-RET* was associated with more aggressive and malignant solid subtypes than the *CCDC6-RET* form by generating *CCDC6-* and *NCOA4-RETs* in Drosophila. Recently, Viswanathan et al. [70] identified an *NCOA4-RET* in low-grade intraductal carcinoma of the salivary gland, the most common intercalated duct type. Paratala. et al. [71] constructed an *NCOA4-RET* fusion protein both in mouse and human cells, and showed a significantly augmented growth ability (i.e., clonogenic expansion) and phosphorylation at tyrosines 905 and 1062 on the RET (the key factors of RET kinase in the MAPK and PI3K-AKT pathways), revealing that MAPK- and PI3K/AKT-signaling pathways participated in a tumorigenic effect mediated by *NCOA4-RET* fusion. Likewise, Chan et al. [64] proved that RET kinase inhibitors and small molecular drugs suppressed the growth of *NCOA4-RET* expressed in NIH/3T3 cells by inhibiting RET phosphorylation and decreasing downstream AKT- and ERK-signaling activities. With a gradual increase in our understanding of the molecular mechanism(s) underlying oncogenic *NCOA4-RET* fusion-mediated tumorigenesis, we expect to see the development of therapies that target NCOA4-mediated tumors.

These essential physiological functions of NCOA4 indicate potential tumorigenic risks, not just the induction of *RET* gene activation. Therefore, additional studies on NCOA4-related functions should provide us with a more comprehensive understanding of the functions of NCOA4.

## Precise regulation of NCOA4 activity

### Precise regulation under physiological conditions

**Fig. 4:**
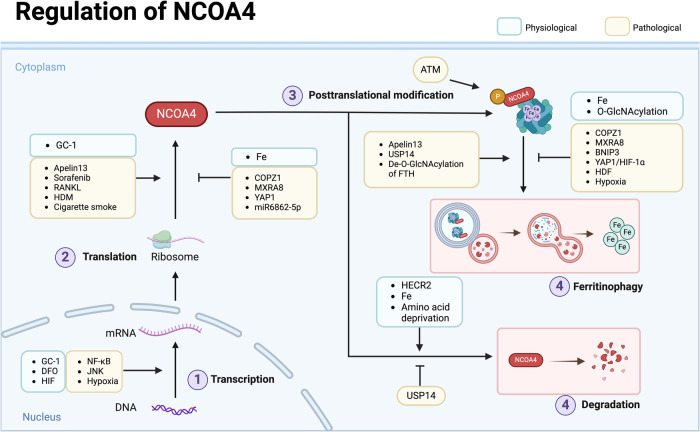
Precise regulation of NCOA4 expression and activity. In both physiological and pathological conditions, NCOA4 is regulated by specific intracellular proteins, small molecular compounds, and various conditions at the levels of transcription, translation, post-translational modification (including ferritinophagy and self-degradation process). The figure illustrates the regulators of NCOA4, using different colors to distinguish physiological and pathological conditions.

As an intracellular iron sensor, NCOA4 can respond sensitively to changes in intracellular iron concentration for homeostatic maintenance under physiological conditions. NCOA4 not only regulates the level of cellular iron but is also regulated by the level of iron. Mancias et al. [23] demonstrated that HERC2 only binds to NCOA4 in a condition of high intracellular iron, mediating NCOA4 breakdown by the proteasome. In contrast, in an iron-depleted condition, HERC2 does not interact with NCOA4. Interestingly, Gao et al. [52] pointed out that elevated expression of NCOA4 mRNA and protein levels were induced by the TR agonist GC-1. However, the gene locus of this interaction remains unclear (Fig. 4).

Li et al. [41] explored the mechanism of NCOA4 mRNA upregulation after undergoing DFO treatment. HIF prolyl hydroxylase domain (PHDs) proteins are oxygen and iron-dependent enzymes that target HIF-α subunits to induce HIF degradation [72]. Chemicals that stabilize HIF such as DFO (as a competitive inhibitor of PHDs) induce the upregulation of NCOA4 expression depending upon the increase in HIF-α. NCOA4 mRNA induction was shown to be weakened by implementing a dual knockdown of HIF-1α and HIF-2α. This work identified a binding site for HIF-1α, HIF-2α, and HIF-1β located at ~1.5 kb upstream of NCOA4 exon 1, in which a hypoxia- response element motif was located at the center.

In addition, Mejlvang et al. [73] reported that amino acid deprivation induced selective autophagy-receptor degradation (including NBR1, TAX1BP1, NDP52 [nuclear domain 10 protein 52], and NCOA4), which was independent of classical macroautophagy, but relied on endosomal sorting complex required for transport (ESCRT)-III component-mediated endosomal microautophagy; this activity required vacuolar protein sorting-associated protein 4 (VPS4) and charged multivesicular body protein 4B. These results reveal a profound effect on NCOA4 under various conditions and provide a novel molecular mechanism for the precise regulation of NCOA4. However, only a few articles entail the regulation of NCOA4 expression, and thus the specific mechanism still needs to be elaborated.

### Precise regulation in the pathological state

Recent studies have shown alterations in NCOA4 expression in various disease states, and some protein-protein interactions can mediate the upregulation of NCOA4 expression (Fig. 4). Ubiquitin-specific peptidase 14 promotes the deubiquitination of NCOA4, inhibiting degradation by ubiquitination and enhancing ferritinophagy, thus mediating ischemic stroke injury [74]. As noted previously, one of the NCOA4 degradation pathways depends on interactions with HERC2. In the neuronal injury model of oxygen-glucose deprivation [17], NCOA4 protein rises, whereas mRNA levels remain unchanged. The protein levels of HERC2 or the combination of HERC2 and NCOA4 were not altered after neuronal injury, indicating that the reduction in NCOA4 ubiquitination in OGD neurons was not due to ubiquitination-promoting enzymes, but rather to the removal of ubiquitin moieties from specific substrate proteins by its competitors. Furthermore, apelin-13, as an endogenous peptide ligand of G protein-coupled receptor angiotensin II protein J [45] effectively induced the expression of sideroflexin1 (SFXN1) and NCOA4 and enhanced the co-localization of NCOA4 and LC3B in a dose- and time-dependent manner [75]. Similarly, house dust mite exposure induced an increase in NCOA4 expression in airway epithelial cells, as well as an elevation in the combination of NCOA4 and FTH1 [76]. Some small molecular chemicals also modulated intracellular NCOA4. Yoshida et al. found that NCOA4 expression levels in lung tissues with COPD (using a cigarette smoke-exposure model) were higher than in normal lung tissues, whereas glutathione peroxidase (GPX) 4 expression levels were diminished. Although the expression changes in NCOA4 are frequently observed in the literature, the mechanism(s) subserving its precise regulation is still unclear.

On the contrary, coatomer protein complex subunit zeta 1 (COPZ1) was identified as a promising target for glioblastoma treatment, involving iron metabolism as a critical component of the COPZ1/NCOA4/FTH1 axis, manifesting a negative regulation of expression and activity of NCOA4. Knockdown of COPZ1 can induce ferritinophagy by increasing NCOA4 and ATG7 expression levels in glioblastoma cells [77]. Similarly, matrix remodeling-associated protein 8 (MXRA8) was also identified as a prognostic indicator for glioma. Knockdown of MXRA8 increased the protein expression of NCOA4 and decreased the levels of FTH1, resulting in ferroptosis of glioma cells [78]. Investigators of one study ascertained that intracellular iron levels increased in BNIP3-depleted cells due to the augmented availability of cytosolic NCOA4 for mediating ferritinophagy, as caused by disturbing BNIP3-NCOA4 interactions [79]. Two recent studies indicated that the expression of NCOA4 was elevated in MLE-12 cells exposed to lipopolysaccharides, whereas overexpression of yes-associated protein [80] 1 attenuated this increase and impaired the interaction between NCOA4 and FTH1 [81, 82].

Some specific conditions can also influence the level of NCOA4. It was shown that a high-fat diet (HFD) led to abnormal hepatic steatosis in mice as well as diminished iron levels, but that it also increased endoplasmic reticulum stress by accumulating p62 and disturbing the expression of NCOA4 and ferritin [83]. As for the influence of hypoxia on NCOA4, this remains under intensive scrutiny. One study showed that hypoxia reduced NCOA4 and increased the levels of mitochondrial ferritin in macrophages [84]. With hypoxia, NCOA4 mRNA fell rapidly, mediated by JNK-enhanced miRNA6862-5p transcription-targeting degradation after 2 h, and reached a significant level after 4 h. Ni et al. reported that HIF-1α inhibited autophagosomal formation to impair autophagic flux under hypoxia, suggesting a negative role for HIF-1α in autophagy [85]. HIF-1α knockout enhanced the free iron pool, promoted mitochondrial iron accumulation, and induced ferroptosis under hypoxia.

Moreover, post-translational modifications can also affect the interaction between NCOA4 and other proteins. Chen and his team discovered that De-O-GlcNAcylate of FTH1 enhanced interactions with NCOA4, regulating ferritinophagy and mitochondrial behaviors to dictate ferroptosis [86]. Recently, Wu et al. [87] creatively identified a phosphorylation modification of NCOA4 at the S550 residue generated by the Ser/Thr protein kinase ATM (a master sensor of DNA damage response, which comprises an indispensable process for ferritinophagy). ATM phosphorylates NCOA4 independent of its classical downstream target transformation-related protein 53 [88]. These findings indicate a novel direction for further study of the precise regulation of NCOA4. However, some questions still need to be resolved. For example, does NCOA4 manifest any other post-translational modifications, and how do these modifications regulate its bioactivity?

## Clinical translation of targeting NCOA4 in diseases

Due to the aforementioned wide range of biological functions intrinsic to NCOA4, many investigators have focused on treatments based directly or indirectly on targeting NCOA4 (Table 1). Inducing ferroptosis of tumor cells via enhancement of NCOA4-mediated ferritinophagy may constitute a potential therapy for cancers. Wang et al. [89] established that vitamin C induced ferritinophagy in 8505C cells, serving as a potential therapeutic agent for cancer. Dihydroartemisinin (DHA) arrests the cell cycle at the G0/G1 phase and inhibits cellular proliferation, inducing AML cell ferroptosis; with DHA’s underlying molecular mechanism(s) reflecting a regulation of the activity of the AMPK/mTOR/p70S6k-signaling pathway to accelerate the degradation of ferritin [90, 91]. The specific inhibitor of O-GlcNAc transferase, OSMI-1, also significantly enhanced cellular sensitivity to ferroptosis by promoting an interaction between FTH1 and NCOA4 [86]. In addition, Zhang et al. [92] revealed that sorafenib treatment upregulated the NCOA4 mRNA-induced increase in autophagosomal generation and macroautophagic flux, initiating ferroptosis in fibrotic hepatocellular carcinoma. These authors subsequently discerned that sorafenib monotherapy also precipitated ZFP36 (an RNA-binding protein that inhibits macroautophagy) downregulation, leading to ferritinophagy and ferroptotic activation in human hepatic stellate cells, ameliorating liver fibrosis [93].

**Table 1:** Pharmacotherapy directly or indirectly targeting NCOA4.

Name	Structure	Mechanism	Efficacy	Cell	Species	Disease	Clinical trial	Reference
**Promote ferroptosis**								
Vitamin C		Induce lipid peroxidation, ferrinophagy activation and ferroptosis (at millimolar concentrations)	Inhibits cancer cells growth and proliferation	Thyroid cancer cell	Human	Anaplastic thyroid cancer	Applicable	Wang, Xu et al. [89]
DHA		1. Arrest the cell cycle	Reduce the cell viability	Leukemia cell	Human	Acute myeloid leukemia	Applicable	Anandhan, Dodson et al., Du, Wang et al. [90, 91]
		2. Induce ferritinophagy						
OSMI-1		1. Decrease the O-GlcNAcylation level	Induce cell death	Cancer cell	Human	Cancer	N/A	Yu et al. [86]
		2. Promote ferritinophagy by accelerating NCOA4-dependent transport of ferritin to lysosomes.						
		3. Enhance the sensitivity to ferroptosis						
Sorafenib		1. Upregulates NCOA4 to induce ferritinophagy-cascade ferroptosis	Inhibit tumor cells growth and metastasis	Cancer cell and Hepatic stellate cell	Human and Mouse	Cancer	Phase IV	Zhang, Yao et al., [92] Zhang, Guo et al. [93]
		2. Downregulate GPX4 to promote ferroptosis						
PDT guided MH-PLGA-IR780 NPs	N/A	1. Increase the expression of NCOA4 while diminishing the expression of ferritin	Promoted cell endocytosis and tumor accumulation in vitro; inhibit tumor growth	Human osteosarcoma cell	Human	Osteosarcoma	N/A	Wang, Zhang et al. [94]
		2. Lead to excessive accumulation of ROS, LPOs, and LipidROS						
		3. Inactivate GPX4 and accumulate ferritinophagy-mediated labile iron						
		4. Cause mitochondrial dysfunction;						
ZnONPs	N/A	Activate the NCOA4-dependent ferritin degradation	Cause vascular inflammation	Endothelial cell	Human and Mouse	Vascular inflammation	N/A	Qin, Zhang et al. [97]
FPBC@SN	N/A	Induce ferritinophagy for ferroptosis	Inhibit tumor cells growth and metastasis	Cancer cell and Fibroblast	Human and Mouse	Cancer, Hepatic fibrosis	N/A	Zuo, Fang et al. [95]
F13A	N/A	Antagonise the increase of NCOA4 induced by apelin-13	Alleviate cardiomyocytes hypertrophy	Cardiomyocyte	Human, Mouse, and Rat	Cardiomyocytes Hypertrophy	N/A	Tang et al. [75]
C3G		Alleviate oxidative stress and inhibit the ferroptosis	Reduced the myocardial infarction	Cardiomyocyte	Rat	Myocardial ischemia/reperfusion injury	Phase III	Shan, Lv et al. [98]
Ferric or ferric agents	N/A	Regulate the expression of NCOA4 and ferritin	Improve insulin resistance and ERS	Liver carcinoma cell	Human and Mouse	Hepatic insulin resistance	Applicable	Jiang et al. [83]
ApoE	N/A	Inhibit ferritinophagy-mediated ferroptosis	Prevent ferroptotic neurodegeneration	N27 neuron	Human	Alzheimer’s disease	N/A	Belaidi, Masaldan et al. [99]
Compound 9a		Disrupt interaction between NCOA4 and FTH1, and decrease the intracellular ferrous iron concentration	Ameliorates the cerebral ischemic-refusion injury	Hippocampal neuronal cell	Human and Rat	Ischemic stroke	N/A	Fang, Chen et al. [100]
Curcumol		Regulate ferritinophagy, alleviate iron overload	Inhibit hepatocyte senescence	Hepatic cell	Mouse and Hamster	NAFLD	Phase III	Qi et al. [100]
NaHS	N/A	Inhibit ferritinophagy and upregulate the expressions of Nrf2 and PPAR-γ	Attenuate PM2.5-induced emphysema and airway inflammation	Human bronchial epithelial cell	Mouse	Emphysema, Airway inflammation	N/A	Wang et al. [82]
HET1006		1. Disrupt the interaction between STING and NCOA4	Remit sepsis and the septic organ injury	PBMC from patients with sepsis and septic mice model	Human and Mouse	Sepsis	N/A	Wu et al. [14]
		2. Decrease the downstream activation of STING, the production of inflammatory cytokines and MDA						

*NCOA4* nuclear receptor coactivator 4, *DHA* Dihydroartemisinin, *N/A* not applicable, *ROS* reactive oxygen species, *LPO* lipid hydroperoxide, *GPX* glutathione peroxidase, *C3G* cyanidin-3-glucoside, *ERS* endoplasmic reticulum stress, *ApoE* apolipoprotein E, *NAFLD* non-alcoholic fatty liver disease, *NaHS* sodium hydrosulfide hydrate, *Nrf2* nuclear factor erythroid2-related factor 2, *PPAR* peroxisome proliferator-activated, *PM* particulate matter, *MDA* malondialdehyde, *STING* stimulator of interferon response cGAMP interactor 1, *PBMC* peripheral blood mononuclear cell.

Nanomedicines have also been widely used clinically in recent years. Photodynamic therapy is a burgeoning technique that encompasses specific drugs (photosensitizers) with light treatment to kill cancer cells. Wang et al. [94] constructed a potential theranostic nanoparticle that consisted of poly(lactic-co-glycolic) acid nanoparticles (NPs) with the fluorescent dye IR780 that showed tumor-targeting ability, and which could induce NCOA4-mediated osteosarcoma cell ferroptosis. Zuo et al. [95] recently invented the FPBC@SN, a type of nanoparticle containing ferritin and a pH-sensitive molecular switch that has been proven to inhibit tumor cell growth and metastasis both in vitro and in vivo. FPBC@SN can release sorafenib when disintegrated in the cytoplasm, upregulating NCOA4 to induce ferritinophagy for ferroptosis. Zinc oxide nanoparticles (ZnONPs) are also considered to possess promising biomedical applications due to their anticancer, antibacterial, antidiabetic, and anti-inflammatory activities; drug-delivery capability; and bioimaging abilities [96]. However, these researchers found that ZnONPs were toxic to the organism. Qin et al. [97] demonstrated that the mechanism underlying ZnONPs-induced ferroptosis was likely associated with the mtROS-AMPK-ULK1-signaling pathway; supplementation with ferrostatin-1 then significantly reversed the vascular injury due to exposure to ZnONPs.

In addition to cancer therapies, most treatment strategies that target ferroptosis attempt to block its effects. F13A, a specific antagonist of the apelin receptor (APJ), can reverse the cardiomyocyte hypertrophy caused by a rise in SFXN1 (a mitochondrial iron-transporting protein) and NCOA4 induced by apelin-13 [75]. Cyanidin-3-glucoside treatment modified oxidative stress, decreased ferroptosis-related protein expression (e.g., in NCOA4), and inhibited the ferroptosis of myocardial cells in myocardial ischemia/reperfusion injury [98]. Targeting NCOA4 also improved abnormal lipid metabolism, as an HFD reduced iron levels, resulting in abnormal hepatic steatosis and insulin resistance. Supplementation with ferric ammonium citrate or ferric agents improved hepatic insulin resistance and ER stress by regulating the expression of NCOA4 and ferritin [83]. Belaidi et al. [99] demonstrated that apolipoprotein E is a potent ferroptosis inhibitor, inhibiting ferritinophagy and averting iron-dependent lipid peroxidation as mediated by the PI3K/AKT-signaling pathway. The small molecular compound 9a, a newly reported ferroptosis inhibitor, reduced intracellular ferrous iron levels by disrupting NCOA4-FTH1 interactions by directly binding to the recombinant protein NCOA4 383 − 522 [100]. There was additional evidence that drugs that targeted NCOA4 delayed cellular aging. For example, curcumol was shown to regulate cellular senescence in treating non-alcoholic fatty liver disease by targeting YAP/NCOA4 [101] suppressing ferritinophagy, alleviating iron overload in hepatic cells, and reducing markers of senescence.

Notably, NCOA4 also plays an essential role in the immunometabolism pathways. In an investigation of emphysema and airway inflammation, pretreatment with exogenous H_2_S (NaHS) attenuated ferroptosis, reducing PM2.5-induced emphysema and airway inflammation by upregulating the expression of nuclear factor erythroid 2- related factor 2 (Nrf2) and PPAR-γ, and inhibiting NCOA4-mediated ferritinophagy, thus restoring redox balance and inhibiting ferroptosis [62]. We recently identified a small molecular compound, HET1006, that mitigated STING-induced ferroptosis both in human cells and a mouse model [14]. As stated above, targeting the regulation of the NCOA4-mediated pathway will contribute to potential treatment strategies that can regulate immunometabolism-related diseases.

## Perspective

In this review, we summarized the various functions of NCOA4 in physiological and pathological processes. NCOA4 participates in various physiological processes based on iron metabolism by orchestrating iron availability, including but not limited to regulating iron mobilization for erythropoiesis, leading to iron overload to catalyze intracellular lipid peroxidation mediating cellular ferroptosis. In addition, its regulatory ability of iron availability also plays a role in host immune modulation, such as affecting NK cell recognition by regulating MHC I expression, influencing bacterial growth, and promoting host cell ferroptosis. On the other hand, the non-ferritinophagic-related functions of NCOA4 are still impossible to ignore, which are based on the study of the structure, including RET-fusion genes for tumorigenesis, co-regulator of nuclear receptors. Several reports have revealed a rare influence of NCOA4 on regulating DNA replication initiation, mitosis, cell growth, proliferation, invasion, etc. Interestingly, some recent studies have pointed out that NCOA4 is extensively involved in innate immunity via iron-dependent and -independent mechanisms. Thus, despite the many milestone studies of the role of NCOA4 in ferritinophagy and ferroptosis, it still cannot obscure the vital role of NCOA4 in immune and metabolism-related pathways.

Future research may shed light on the different functions of conversion of NCOA4 during diseases, such as the disturbance between nuclear function (co-regulator of nuclear receptors) and cytoplasmic function (iron metabolism and immune regulation) in different stress conditions. In addition, copper, magnesium, and iron have similar biological and physical features and sometimes share the same chelating protein. Thus, exploring whether NCOA4 can sense and regulate other similar trace elements metabolism will be interesting. Finally, it is still unknown whether there are other post-translational modifications and the roles and functions of these modifications. Based on the investigation of the above issues, the multifunctional effects of NCOA4 propose that chemical regulation might be a potentially novel therapeutic strategy for various heterogeneous diseases.

## References

[1] Mancias JD, Wang X, Gygi SP, Harper JW, Kimmelman AC (2014). Quantitative proteomics identifies NCOA4 as the cargo receptor mediating ferritinophagy. Nature.

[2] Hou W, Xie Y, Song X, Sun X, Lotze MT, Zeh HJ (2016). Autophagy promotes ferroptosis by degradation of ferritin. Autophagy.

[3] Gao M, Monian P, Pan Q, Zhang W, Xiang J, Jiang X (2016). Ferroptosis is an autophagic cell death process. Cell Res.

[4] Dai Y, Zhu C, Xiao W, Chen X, Cai Y. Mycobacterium tuberculosis induces host autophagic ferritin degradation for enhanced iron bioavailability and bacterial growth. Autophagy. 2023;1–3. 10.1080/15548627.2023.2213983. 10.1080/15548627.2023.2213983 PMC11062391 37198940

[5] Dai Y, Zhu C, Xiao W, Huang K, Wang X, Shi C, et al. Mycobacterium tuberculosis hijacks host TRIM21- and NCOA4-dependent ferritinophagy to enhance intracellular growth. J Clin Investig. 2023;133. 10.1172/JCI159941. 10.1172/JCI159941 PMC10104892 37066876

[6] Federico G, Carrillo F, Dapporto F, Chiariello M, Santoro M, Bellelli R (2022). NCOA4 links iron bioavailability to DNA metabolism. Cell Rep.

[7] Santana-Codina N, Del Rey MQ, Kapner KS, Zhang H, Gikandi A, Malcolm C (2022). NCOA4-mediated ferritinophagy is a pancreatic cancer dependency via maintenance of iron bioavailability for iron-sulfur cluster proteins. Cancer Discov.

[8] Jin L, Yu B, Wang H, Shi L, Yang J, Wu L (2023). STING promotes ferroptosis through NCOA4-dependent ferritinophagy in acute kidney injury. Free Radic Biol Med.

[9] Santoro M, Dathan NA, Berlingieri MT, Bongarzone I, Paulin C, Grieco M (1994). Molecular characterization of RET/PTC3; a novel rearranged version of the RETproto-oncogene in a human thyroid papillary carcinoma. Oncogene.

[10] Yeh S, Chang C (1996). Cloning and characterization of a specific coactivator, ARA70, for the androgen receptor in human prostate cells. Proc Natl Acad Sci USA.

[11] Kollara A, Brown TJ (2010). Variable expression of nuclear receptor coactivator 4 (NcoA4) during mouse embryonic development. J Histochem Cytochem.

[12] Viladomiu M, Hontecillas R, Pedragosa M, Carbo A, Hoops S, Michalak P (2012). Modeling the role of peroxisome proliferator-activated receptor gamma and microRNA-146 in mucosal immune responses to Clostridium difficile. PLoS One.

[13] Sottile R, Federico G, Garofalo C, Tallerico R, Faniello MC, Quaresima B (2019). Iron and ferritin modulate MHC class I expression and NK cell recognition. Front Immunol.

[14] Wu J, Liu Q, Zhang X, Tan M, Li X, Liu P (2022). The interaction between STING and NCOA4 exacerbates lethal sepsis by orchestrating ferroptosis and inflammatory responses in macrophages. Cell Death Dis.

[15] Strausberg RL, Feingold EA, Grouse LH, Derge JG, Klausner RD, Collins FS (2002). Generation and initial analysis of more than 15,000 full-length human and mouse cDNA sequences. Proc Natl Acad Sci USA.

[16] Kollara A, Brown TJ (2012). Expression and function of nuclear receptor co-activator 4: evidence of a potential role independent of co-activator activity. Cell Mol Life Sci.

[17] Bogdan AR, Miyazawa M, Hashimoto K, Tsuji Y (2016). Regulators of iron homeostasis: new players in metabolism, cell death, and disease. Trends Biochem Sci.

[18] Muckenthaler MU, Rivella S, Hentze MW, Galy B (2017). A red carpet for iron metabolism. Cell.

[19] Pantopoulos K, Porwal SK, Tartakoff A, Devireddy L (2012). Mechanisms of mammalian iron homeostasis. Biochemistry.

[20] Camaschella C (2017). New insights into iron deficiency and iron deficiency anemia. Blood Rev.

[21] Weis S, Carlos AR, Moita MR, Singh S, Blankenhaus B, Cardoso S (2017). Metabolic adaptation establishes disease tolerance to sepsis. Cell.

[22] Dowdle WE, Nyfeler B, Nagel J, Elling RA, Liu S, Triantafellow E (2014). Selective VPS34 inhibitor blocks autophagy and uncovers a role for NCOA4 in ferritin degradation and iron homeostasis in vivo. Nat Cell Biol.

[23] Mancias JD, Pontano Vaites L, Nissim S, Biancur DE, Kim AJ, Wang X, et al. Ferritinophagy via NCOA4 is required for erythropoiesis and is regulated by iron dependent HERC2-mediated proteolysis. Elife. 2015;4. 10.7554/eLife.10308. 10.7554/eLife.10308 PMC4592949 26436293

[24] Kimura T, Jia J, Kumar S, Choi SW, Gu Y, Mudd M (2017). Dedicated SNAREs and specialized TRIM cargo receptors mediate secretory autophagy. EMBO J.

[25] De Domenico I, Ward DM, Kaplan J (2009). Specific iron chelators determine the route of ferritin degradation. Blood.

[26] Asano T, Komatsu M, Yamaguchi-Iwai Y, Ishikawa F, Mizushima N, Iwai K (2011). Distinct mechanisms of ferritin delivery to lysosomes in iron-depleted and iron-replete cells. Mol Cell Biol.

[27] Gryzik M, Srivastava A, Longhi G, Bertuzzi M, Gianoncelli A, Carmona F (2017). Expression and characterization of the ferritin binding domain of Nuclear Receptor Coactivator-4 (NCOA4). Biochim Biophys Acta Gen Subj.

[28] Srivastava AK, Flint N, Kreckel H, Gryzik M, Poli M, Arosio P (2020). Thermodynamic and kinetic studies of the interaction of nuclear receptor coactivator-4 (NCOA4) with human ferritin. Biochemistry.

[29] Kuno S, Fujita H, Tanaka YK, Ogra Y, Iwai K (2022). Iron-induced NCOA4 condensation regulates ferritin fate and iron homeostasis. EMBO Rep.

[30] Goodwin JM, Dowdle WE, DeJesus R, Wang Z, Bergman P, Kobylarz M (2017). Autophagy-independent lysosomal targeting regulated by ULK1/2-FIP200 and ATG9. Cell Rep.

[31] Truman-Rosentsvit M, Berenbaum D, Spektor L, Cohen LA, Belizowsky-Moshe S, Lifshitz L (2018). Ferritin is secreted via 2 distinct nonclassical vesicular pathways. Blood.

[32] Cohen LA, Gutierrez L, Weiss A, Leichtmann-Bardoogo Y, Zhang DL, Crooks DR (2010). Serum ferritin is derived primarily from macrophages through a nonclassical secretory pathway. Blood.

[33] Bellelli R, Federico G, Matte A, Colecchia D, Iolascon A, Chiariello M (2016). NCOA4 deficiency impairs systemic iron homeostasis. Cell Rep.

[34] Yanatori I, Richardson DR, Dhekne HS, Toyokuni S, Kishi F (2021). CD63 is regulated by iron via the IRE-IRP system and is important for ferritin secretion by extracellular vesicles. Blood.

[35] Xu J, Yang KC, Go NE, Colborne S, Ho CJ, Hosseini-Beheshti E (2022). Chloroquine treatment induces secretion of autophagy-related proteins and inclusion of Atg8-family proteins in distinct extracellular vesicle populations. Autophagy.

[36] Weber GJ, Choe SE, Dooley KA, Paffett-Lugassy NN, Zhou Y, Zon LI (2005). Mutant-specific gene programs in the zebrafish. Blood.

[37] Nilsson R, Schultz IJ, Pierce EL, Soltis KA, Naranuntarat A, Ward DM (2009). Discovery of genes essential for heme biosynthesis through large-scale gene expression analysis. Cell Metab.

[38] Ryu MS, Zhang D, Protchenko O, Shakoury-Elizeh M, Philpott CC (2017). PCBP1 and NCOA4 regulate erythroid iron storage and heme biosynthesis. J Clin Invest.

[39] Santana-Codina N, Gableske S, Quiles del Rey M, Malachowska B, Jedrychowski MP, Biancur DE (2019). NCOA4 maintains murine erythropoiesis via cell autonomous and non-autonomous mechanisms. Haematologica.

[40] Philpott CC (2020). Iron on the move: mobilizing liver iron via NCOA4. Blood.

[41] Li X, Lozovatsky L, Sukumaran A, Gonzalez L, Jain A, Liu D (2020). NCOA4 is regulated by HIF and mediates mobilization of murine hepatic iron stores after blood loss. Blood.

[42] Nai A, Lidonnici MR, Federico G, Pettinato M, Olivari V, Carrillo F (2021). NCOA4-mediated ferritinophagy in macrophages is crucial to sustain erythropoiesis in mice. Haematologica.

[43] Dixon SJ, Lemberg KM, Lamprecht MR, Skouta R, Zaitsev EM, Gleason CE (2012). Ferroptosis: an iron-dependent form of nonapoptotic cell death. Cell.

[44] Liang D, Feng Y, Zandkarimi F, Wang H, Zhang Z, Kim J (2023). Ferroptosis surveillance independent of GPX4 and differentially regulated by sex hormones. Cell.

[45] Tatemoto K, Hosoya M, Habata Y, Fujii R, Kakegawa T, Zou MX (1998). Isolation and characterization of a novel endogenous peptide ligand for the human APJ receptor. Biochem Biophys Res Commun.

[46] Kollara A, Ringuette MJ, Brown TJ (2011). Dynamic distribution of nuclear coactivator 4 during mitosis: association with mitotic apparatus and midbodies. PLoS One.

[47] Yeh S, Miyamoto H, Shima H, Chang C (1998). From estrogen to androgen receptor: a new pathway for sex hormones in prostate. Proc Natl Acad Sci USA.

[48] Alen P, Claessens F, Schoenmakers E, Swinnen JV, Verhoeven G, Rombauts W (1999). Interaction of the putative androgen receptor-specific coactivator ARA70/ELE1alpha with multiple steroid receptors and identification of an internally deleted ELE1beta isoform. Mol Endocrinol.

[49] Lanzino M, De Amicis F, McPhaul MJ, Marsico S, Panno ML, Ando S (2005). Endogenous coactivator ARA70 interacts with estrogen receptor alpha (ERalpha) and modulates the functional ERalpha/androgen receptor interplay in MCF-7 cells. J Biol Chem.

[50] Ting HJ, Bao BY, Hsu CL, Lee YF (2005). Androgen-receptor coregulators mediate the suppressive effect of androgen signals on vitamin D receptor activity. Endocrine.

[51] Privalsky ML (2004). The role of corepressors in transcriptional regulation by nuclear hormone receptors. Annu Rev Physiol.

[52] Gao X, Lee HY, Li W, Platt RJ, Barrasa MI, Ma Q (2017). Thyroid hormone receptor beta and NCOA4 regulate terminal erythrocyte differentiation. Proc Natl Acad Sci USA.

[53] Wahli W, Michalik L (2012). PPARs at the crossroads of lipid signaling and inflammation. Trends Endocrinol Metab.

[54] Lazar MA (2005). PPAR gamma, 10 years later. Biochimie.

[55] Heinlein CA, Chang C (2003). Induction and repression of peroxisome proliferator-activated receptor alpha transcription by coregulator ARA70. Endocrine.

[56] Yao L, Hou J, Wu X, Lu Y, Jin Z, Yu Z (2023). Cancer-associated fibroblasts impair the cytotoxic function of NK cells in gastric cancer by inducing ferroptosis via iron regulation. Redox Biol.

[57] Bauckman KA, Mysorekar IU (2016). Ferritinophagy drives uropathogenic Escherichia coli persistence in bladder epithelial cells. Autophagy.

[58] Paules CI, Marston HD, Bloom ME, Fauci AS (2018). Tickborne diseases—confronting a growing threat. N Engl J Med.

[59] Yan Q, Zhang W, Lin M, Teymournejad O, Budachetri K, Lakritz J, et al. Iron robbery by intracellular pathogen via bacterial effector-induced ferritinophagy. Proc Natl Acad Sci USA 2021;118. 10.1073/pnas.2026598118. 10.1073/pnas.2026598118 PMC8201858 34074773

[60] Sun Y, Bao Q, Xuan B, Xu W, Pan D, Li Q, et al. Human cytomegalovirus protein pUL38 prevents premature cell death by binding to ubiquitin-specific protease 24 and regulating iron metabolism. J Virol. 2018;92. 10.1128/JVI.00191-18. 10.1128/JVI.00191-18 PMC6002719 29695420

[61] Liu Q, Wu J, Zhang X, Li X, Wu X, Zhao Y (2021). Circulating mitochondrial DNA-triggered autophagy dysfunction via STING underlies sepsis-related acute lung injury. Cell Death Dis.

[62] Wang Y, Liao S, Pan Z, Jiang S, Fan J, Yu S (2022). Hydrogen sulfide alleviates particulate matter-induced emphysema and airway inflammation by suppressing ferroptosis. Free Radic Biol Med.

[63] Han L, Bai L, Qu C, Dai E, Liu J, Kang R (2021). PPARG-mediated ferroptosis in dendritic cells limits antitumor immunity. Biochem Biophys Res Commun.

[64] Chan AW, Pan Y, Tong JH, Lung RW, Kwan JS, Chow C (2020). Receptor tyrosine kinase fusions act as a significant alternative driver of the serrated pathway in colorectal cancer development. J Pathol.

[65] Kollara A, Kahn HJ, Marks A, Brown TJ (2001). Loss of androgen receptor associated protein 70 (ARA70) expression in a subset of HER2-positive breast cancers. Breast Cancer Res Treat.

[66] Ligr M, Li Y, Zou X, Daniels G, Melamed J, Peng Y (2010). Tumor suppressor function of androgen receptor coactivator ARA70alpha in prostate cancer. Am J Pathol.

[67] Bellelli R, Castellone MD, Guida T, Limongello R, Dathan NA, Merolla F (2014). NCOA4 transcriptional coactivator inhibits activation of DNA replication origins. Mol Cell.

[68] Yang SR, Aypar U, Rosen EY, Mata DA, Benayed R, Mullaney K (2021). A performance comparison of commonly used assays to detect RET fusions. Clin Cancer Res.

[69] Levinson S, Cagan RL (2016). Drosophila cancer models identify functional differences between ret fusions. Cell Rep.

[70] Viswanathan K, Sadow PM, Maleki Z, Nishino M, Baloch ZW, Abbott TE (2021). Cytomorphologic features of intraductal salivary gland carcinoma: A multi-institutional study of 13 FNA cases with histologic, molecular, and clinical correlations. Cancer Cytopathol.

[71] Paratala BS, Chung JH, Williams CB, Yilmazel B, Petrosky W, Williams K (2018). RET rearrangements are actionable alterations in breast cancer. Nat Commun.

[72] Bailey PSJ, Nathan JA. Metabolic regulation of hypoxia-inducible transcription factors: the role of small molecule metabolites and iron. Biomedicines. 2018;6. 10.3390/biomedicines6020060. 10.3390/biomedicines6020060 PMC6027492 29772792

[73] Mejlvang J, Olsvik H, Svenning S, Bruun JA, Abudu YP, Larsen KB (2018). Starvation induces rapid degradation of selective autophagy receptors by endosomal microautophagy. J Cell Biol.

[74] Li C, Sun G, Chen B, Xu L, Ye Y, He J (2021). Nuclear receptor coactivator 4-mediated ferritinophagy contributes to cerebral ischemia-induced ferroptosis in ischemic stroke. Pharm Res.

[75] Tang M, Huang Z, Luo X, Liu M, Wang L, Qi Z (2019). Ferritinophagy activation and sideroflexin1-dependent mitochondria iron overload is involved in apelin-13-induced cardiomyocytes hypertrophy. Free Radic Biol Med.

[76] Zeng Z, Huang H, Zhang J, Liu Y, Zhong W, Chen W (2022). HDM induce airway epithelial cell ferroptosis and promote inflammation by activating ferritinophagy in asthma. FASEB J.

[77] Zhang Y, Kong Y, Ma Y, Ni S, Wikerholmen T, Xi K (2021). Loss of COPZ1 induces NCOA4 mediated autophagy and ferroptosis in glioblastoma cell lines. Oncogene.

[78] Xu Z, Chen X, Song L, Yuan F, Yan Y (2022). Matrix remodeling-associated protein 8 as a novel indicator contributing to glioma immune response by regulating ferroptosis. Front Immunol.

[79] Vara-Perez M, Rossi M, Van den Haute C, Maes H, Sassano ML, Venkataramani V (2021). BNIP3 promotes HIF-1alpha-driven melanoma growth by curbing intracellular iron homeostasis. EMBO J.

[80] Kashyap AS, Fernandez-Rodriguez L, Zhao Y, Monaco G, Trefny MP, Yoshida N (2019). GEF-H1 signaling upon microtubule destabilization is required for dendritic cell activation and specific anti-tumor responses. Cell Rep.

[81] Zhang J, Zheng Y, Wang Y, Wang J, Sang A, Song X (2022). YAP1 alleviates sepsis-induced acute lung injury via inhibiting ferritinophagy-mediated ferroptosis. Front Immunol.

[82] Wang J, Zhu Q, Li R, Zhang J, Ye X, Li X (2022). YAP1 protects against septic liver injury via ferroptosis resistance. Cell Biosci.

[83] Jiang C, Zhang S, Li D, Chen L, Zhao Y, Mei G (2020). Impaired ferritinophagy flux induced by high fat diet mediates hepatic insulin resistance via endoplasmic reticulum stress. Food Chem Toxicol.

[84] Fuhrmann DC, Mondorf A, Beifuss J, Jung M, Brune B (2020). Hypoxia inhibits ferritinophagy, increases mitochondrial ferritin, and protects from ferroptosis. Redox Biol.

[85] Ni S, Yuan Y, Qian Z, Zhong Z, Lv T, Kuang Y (2021). Hypoxia inhibits RANKL-induced ferritinophagy and protects osteoclasts from ferroptosis. Free Radic Biol Med.

[86] Yu F, Zhang Q, Liu H, Liu J, Yang S, Luo X (2022). Dynamic O-GlcNAcylation coordinates ferritinophagy and mitophagy to activate ferroptosis. Cell Discov.

[87] Blackford AN, Jackson SP (2017). ATM, ATR, and DNA-PK: the trinity at the heart of the DNA damage response. Mol Cell.

[88] Wu H, Liu Q, Shan X, Gao W, Chen, Q. ATM orchestrates ferritinophagy and ferroptosis by phosphorylating NCOA4. Autophagy. 2023;1–16. 10.1080/15548627.2023.2170960. 10.1080/15548627.2023.2170960 PMC10283418 36752571

[89] Wang X, Xu S, Zhang L, Cheng X, Yu H, Bao J (2021). Vitamin C induces ferroptosis in anaplastic thyroid cancer cells by ferritinophagy activation. Biochem Biophys Res Commun.

[90] Anandhan A, Dodson M, Shakya A, Chen J, Liu P, Wei Y (2023). NRF2 controls iron homeostasis and ferroptosis through HERC2 and VAMP8. Sci Adv.

[91] Du J, Wang T, Li Y, Zhou Y, Wang X, Yu X (2019). DHA inhibits proliferation and induces ferroptosis of leukemia cells through autophagy dependent degradation of ferritin. Free Radic Biol Med.

[92] Zhang Z, Yao Z, Wang L, Ding H, Shao J, Chen A (2018). Activation of ferritinophagy is required for the RNA-binding protein ELAVL1/HuR to regulate ferroptosis in hepatic stellate cells. Autophagy.

[93] Zhang Z, Guo M, Li Y, Shen M, Kong D, Shao J (2020). RNA-binding protein ZFP36/TTP protects against ferroptosis by regulating autophagy signaling pathway in hepatic stellate cells. Autophagy.

[94] Wang Y, Zhang L, Zhao G, Zhang Y, Zhan F, Chen Z (2022). Homologous targeting nanoparticles for enhanced PDT against osteosarcoma HOS cells and the related molecular mechanisms. J Nanobiotechnology.

[95] Zuo T, Fang T, Zhang J, Yang J, Xu R, Wang Z (2021). pH-sensitive molecular-switch-containing polymer nanoparticle for breast cancer therapy with ferritinophagy-cascade ferroptosis and tumor immune activation. Adv Health Mater.

[96] Xiong HM (2013). ZnO nanoparticles applied to bioimaging and drug delivery. Adv Mater.

[97] Qin X, Zhang J, Wang B, Xu G, Yang X, Zou Z (2021). Ferritinophagy is involved in the zinc oxide nanoparticles-induced ferroptosis of vascular endothelial cells. Autophagy.

[98] Shan X, Lv ZY, Yin MJ, Chen J, Wang J, Wu QN (2021). The protective effect of cyanidin-3-glucoside on myocardial ischemia-reperfusion injury through ferroptosis. Oxid Med Cell Longev.

[99] Belaidi AA, Masaldan S, Southon A, Kalinowski P, Acevedo K, Appukuttan AT, et al. Apolipoprotein E potently inhibits ferroptosis by blocking ferritinophagy. Mol Psychiatry. 2022. 10.1038/s41380-022-01568-w. 10.1038/s41380-022-01568-w PMC9757994 35484240

[100] Fang Y, Chen X, Tan Q, Zhou H, Xu J, Gu Q (2021). Inhibiting ferroptosis through disrupting the NCOA4-FTH1 interaction: a new mechanism of action. ACS Cent Sci.

[101] Qi X, Song A, Ma M, Wang P, Zhang X, Lu C (2021). Curcumol inhibits ferritinophagy to restrain hepatocyte senescence through YAP/NCOA4 in non-alcoholic fatty liver disease. Cell Prolif.

